# Circulating Nrf2, Glutathione, and Malondialdehyde Correlate with Disease Severity in Duchenne Muscular Dystrophy

**DOI:** 10.3390/antiox12040871

**Published:** 2023-04-03

**Authors:** Tomas Almeida-Becerril, Maricela Rodríguez-Cruz, Judith Villa-Morales, Christian Ricardo Sánchez-Mendoza, Jose Emilio Galeazzi-Aguilar

**Affiliations:** 1 Laboratorio de Nutrición Molecular, Unidad de Investigación Médica en Nutrición, Unidad Médica de Alta Especialidad Hospital de Pediatría “Dr. Silvestre Frenk Freund”, Centro Médico Nacional Siglo XXI, Instituto Mexicano del Seguro Social (IMSS), Mexico City 06725, Mexico; 2 Posgrado en Ciencias Biológicas, Universidad Nacional Autónoma de México (UNAM), Mexico City 04510, Mexico; 3 Departamento de Genética, Unidad Médica de Alta Especialidad Hospital General “Dr. Gaudencio González Garza”, Centro Médico Nacional La Raza, Instituto Mexicano del Seguro Social(IMSS), Mexico City 02990, Mexico; 4 Departamento de Genética Médica, Hospital de Pediatría “Dr. Silvestre Frenk Freund”, Centro Médico Nacional Siglo XXI, IMSS, Mexico City 06725, Mexico

**Keywords:** Nrf2, glutathione, MDA, protein carbonyl, Duchenne muscular dystrophy

## Abstract

Oxidative stress (OS) plays an essential role in the pathophysiology of Duchenne muscular dystrophy (DMD). However, the actors that regulate OS need to be better studied. We aimed to evaluate whether NFE2-like bZIP transcription factor 2 (Nrf2), glutathione, malondialdehyde (MDA), and protein carbonyl concentrations change according to the disease severity in DMD patients. Moreover, we assessed whether OS correlated with muscle injury, clinical characteristics, physical activity, and antioxidant food consumption (AFC). A total of 28 DMD patients participated in this study. OS markers, metabolic indicators, and enzymatic markers of muscle injury were measured in circulation. Muscle injury was measured with clinical scales, and physical activity and AFC were evaluated with questionnaires. Nrf2 concentration was lower (*p ≤* 0.01), and malondialdehyde concentration was higher (*p* < 0.05) in non-ambulatory patients than in ambulatory patients. Nrf2 correlated with age (*rho* = −0.387), Vignos scale (*rho* = −0.328), GMFCS scale (*rho* = −0.399), and Brooke scale scores (*rho* = −0.371) (*p* < 0.05). MDA correlated with Vignos (*rho* = 0.317) and Brooke scale scores (*rho* = 0.414) (*p* ≤ 0.05). In conclusion, DMD patients with the worst muscle function had more significant oxidative damage and lower antioxidant function than DMD patients with better muscle function.

## 1. Introduction

Duchenne muscular dystrophy (DMD) is characterized by weakness and chronic muscle injury evidenced by a circulating increase of creatine phosphokinase (CPK), alanine aminotransferase (ALT), and aspartate aminotransferase (AST) [1]. DMD is caused by mutations in the *DMD* gene resulting in the absence of protein dystrophin [2,3,4]. The lack of dystrophin triggers progressive muscle wasting exacerbated by hyper-inflammation, pro-fibrotic response, and elevated reactive oxygen species (ROS) production [5,6]. Whether ROS production exceeds the physiological antioxidant capacity, oxidative stress (OS) is triggered, leading to oxidative damage [7]. It is well known that ROS are harmful to all tissues, but dystrophic muscle is more susceptible to cell death [8].

Numerous studies have reported OS markers in the circulation of DMD patients. However, markers such as master factor NFE2-like bZIP transcription factor 2 (Nrf2), glutathione, malondialdehyde (MDA), and protein carbonyl have been poorly or non-studied in different stages of muscle injury in dystrophic patients.

Nrf2 regulates OS through its antioxidant function by interacting with the antioxidant-responsive element sequence to control the expression of antioxidant enzymes (glutathione S-transferase and NAD(P)H: quinone oxidoreductase) [9]. The scarce evidence has demonstrated higher levels of NRF2 mRNA in muscle biopsies of DMD patients compared with healthy controls. These levels are lower in older patients (2–9 years of age) than in younger patients (0–2 years of age) [10]. Consistently, we previously reported that levels of NRF2 mRNA from circulating leucocytes tend to be lower in non-ambulatory DMD patients than in ambulatory DMD patients [11]. The above evidence suggests a role of Nrf2 in the disease severity. Therefore, studies of Nrf2 circulating protein are crucial to support this hypothesis.

Glutathione is another antioxidant player detected in muscle biopsy of DMD patients, but the information about its concentration needs more studies. For instance, Petrillo et al. [10] reported that glutathione concentration is lower in muscle biopsies from DMD patients compared with healthy controls. However, Burns et al. [12] did not observe differences in the glutathione concentration in diaphragm muscle from adult mdx mice (DMD model) compared with healthy control mice. Moreover, although glutathione concentration is higher in the muscle of older DMD patients (0–2 vs. 2–9 years of age), changes in circulation were not detected [10].

Oxidative damage in DMD has been elucidated in mdx mice and patients. For instance, markers of lipid peroxidation (LPX), such as PF2-Isoprostane, thiobarbituric acid-reactive substances (TBARS), and conjugated dienes, are elevated in muscle biopsy and plasma from DMD patients compared with healthy controls [13,14,15]. Additionally, we reported that circulating MDA and 8-isoprostane concentrations are increased in non-ambulatory DMD patients compared with those ambulatories. Those markers correlate with a muscle injury, suggesting a relation between LPX and disease severity [11].

Regarding protein carbonyl, this oxidative damage marker was found elevated in the whole blood of DMD patients (0–9 years of age) compared with healthy controls [10]. Consistently, in the mdx model, this marker was higher in plasma at 18 months than in healthy controls. However, no differences were observed when concentrations were compared between muscle samples of the mdx model and healthy mice [16].

OS plays a role in the pathophysiology of DMD; however, factors influencing it have not been thoroughly studied. It is well known that high body mass index (BMI), physical activity, metabolic indicators, and diet are associated with OS [17]. Therefore, further studies are needed to know whether a relationship exists between circulating OS markers concentration and the severity of the disease, considering factors that influence it. Studying OS in different stages of the disease is essential to improve the knowledge of DMD pathophysiology.

Additionally, studying body fluids such as blood in this context is necessary since access to human tissues is limited and invasive, mainly in pediatric patients. Therefore, we aimed to evaluate whether circulating antioxidant (Nrf2 and glutathione) and oxidative damage markers (MDA and protein carbonyl) concentration changes according to disease severity in DMD patients. Also, we assessed whether these players of OS correlate with muscle injury markers, BMI, metabolic indicators, physical activity, and AFC. We evidenced in this work that DMD patients with lower muscle function have more significant oxidative damage and a lower antioxidant function than DMD patients with better muscle function.

## 2. Materials and Methods

### 2.1. Patients

This was a prospective cross-sectional study. A total of 28 children with DMD were recruited from the Pediatric Hospital of the Centro Médico Nacional Siglo XXI, Instituto Mexicano del Seguro Social (IMSS) and the General Hospital of the Centro Médico Nacional La Raza, IMSS. Inclusion criteria for DMD patients were (a) clinical and molecular diagnosis of DMD, (b) age from 3 to 15 years of age, (c) corticosteroid naïve, and (d) no intake of antioxidants supplements. The research was conducted at the Laboratory of Molecular Nutrition from Medical Research Unit in Nutrition at the IMSS. The study was approved by the Institutional Ethics Committee of Scientific Research of IMSS (R-2018-785-089). All parents provided written informed consent to participate in the study following the Declaration of Helsinki of 1975 and the Human Subjects’ Guidelines of the IMSS Institutional Ethics Committee before participating.

### 2.2. Procedures

On the study day, a sample of 6 mL of peripheral blood was collected from all the subjects by venous puncture (after a 12 h overnight fast) in a Vacutainer^TM^ EDTA and a Vacutainer^TM^ serum separator gel. Plasma and serum were separated by centrifugation and stored at −70 °C to quantify circulating markers of OS in plasma, muscle injury enzymes, and metabolic parameters in serum. Then, age, muscle injury, and anthropometric parameters were registered. Physical activity was registered in a questionnaire, and a Food Frequency Questionnaire (FFQ) was applied. Patients were classified according to ambulatory status (ambulatory and non-ambulatory patients).

### 2.3. Measurements

#### 2.3.1. Circulating Markers of Oxidative Stress: Antioxidant Response

We measured Nrf2 concentration with the RayBio^®^ Human Nrf2 ELISA Kit (RayBiotech Life, Inc, Peachtree Corners, GA, USA) and glutathione concentration with the Glutathione Colorimetric Assay Kit (Cayman Chemical Company, Ann Arbor, MI, USA) in plasma. All samples and standards were measured in duplicate.

#### 2.3.2. Circulating Markers of Oxidative Stress: Oxidative Damage

The lipid peroxidation marker MDA was measured with the TBARS (TCA Method) Colorimetric Assay Kit (Cayman Chemical Company, Ann Arbor, MI, USA), and the protein oxidation was measured with the Protein Carbonyl Colorimetric Assay Kit (Cayman Chemical Company, Ann Arbor, MI, USA) in plasma. All samples and standards were measured in duplicate.

#### 2.3.3. Muscle Injury Markers

Muscle injury was evaluated in terms of function and strength using clinical scales [11,18] and quantifying circulating markers of muscle injury. The muscle function of the lower limbs was graded using the Vignos scale [19] and the Gross Motor Function Classification System (GMFCS) [20]. The muscle function of the upper limbs was graded using the Brooke scale [21]. The muscle strength was evaluated using the Medical Research Council (MRC) scale [22].

Regarding the circulating markers of muscle injury, CPK (U/L) was determined in serum via chemiluminescent immunometric assay using a commercial kit (Spinreact, Girona, Spain). ALT and AST (U/L) were determined in serum with enzymatic-colorimetric kits (Spinreact Girona, Spain). All reactions (CPK, ALT, and AST) were performed in a clinical analyzer SPIN 120 (Spinreact, Girona, Spain).

#### 2.3.4. Anthropometric Parameters

Trained nutriologists carried out the anthropometric measurements. Height (cm) was measured with a wall-mounted roll-up measuring tape Seca^®^ in ambulatory patients and the height in non-ambulatory patients with the summation-of-body-parts method in a supine position using an ergonomic tape Seca^®^ 201. The Body weight (kg) was measured using a Seca^®^ 954 wheelchair-bound scale for ambulatory and non-ambulatory patients. The body mass index (BMI) percentile was calculated using growth charts for boys (2–20 years of age) from the Centers for Disease Control and Prevention.

#### 2.3.5. Metabolic Indicators

Serum glucose (mg/dL), triglycerides (mg/dL), and total cholesterol (mg/dL) concentrations were quantified by enzymatic assays in a clinical analyzer SPIN 120 (Spinreact, Girona, Spain). Very-low-density lipoprotein-cholesterol (VLDL-C) concentration was estimated with the Friedewald equation [23].

#### 2.3.6. Physical Activity

Physical activity was evaluated according to two parameters: the frequency of therapy sessions (sessions/week) and the current physical activity. The last was evaluated with the Physical Activity Questionnaire (PAQ-C) [24] that we previously adapted for Mexican boys with DMD. PAQ-C is a seven-day recall instrument composed of nine items graded on a five-point scale. The questionnaire was applied face-to-face to parents or legal guardians and patients. For a successful application, we explained to parents and patients that PAQ-C was not a test and emphasized that we were interested in registering the activity during the last seven days. The final PAQ-C activity score is obtained from the mean of the nine items. The final score ranges from 1 to 5, where 1 indicates low physical activity and 5 indicates high physical activity.

#### 2.3.7. Frequency of Antioxidant Food Consumption

We evaluated the diet of DMD patients considering the frequency of the antioxidant food consumption (AFC) approach. For this purpose, the FFQ, previously designed by the National Institute of Public Health of Mexico, was applied face-to-face by a trained nutritionist [25,26].

During the administration, parents and patients were asked to recall the frequency of consumption of 188 foods (items) categorized by food groups: 23 vegetables, 27 fruits, 14 milk types or milk products, 9 fish or seafood, 14 types of red meat, 9 types of white meat, 7 legumes, 26 kinds of cereals and tubers, 19 animal and vegetable fats, 15 sugars and confectionery, 12 beverages, and 13 traditional or fast food. Then, we selected only the foods with the highest antioxidant capacity considering the antioxidant capacity of food reported by Carlsen et al. [27]. Finally, the frequency of consumption was calculated by converting to times/day as follows: never or a few times/year was converted to 0 times/day, one time/month to 0.033 times/day, 2–3 times/month to 0.083 times/day, one time/week to 0.143 times/day, 2–4 times/week to 0.429 times/day, 5–6 times/week to 0.786 times/day, one time/day remained the same, and two or more times/day to 2 times/day.

### 2.4. Statistical Analyses

The SPSS statistical software (SPSS ver. 24, Chicago, IL, USA) was used for statistical analyses. Variables distribution was evaluated with the Shapiro-Wilk test. The data were presented as mean ± standard deviation (SD) when they followed a normal distribution or median (minimum, maximum) when they followed a non-normal distribution. Comparisons between ambulatory and non-ambulatory patients were performed by Student *t*-test for normal data or Mann-Whitney *U*-test for non-normal data. Correlations between variables were estimated with Spearman’s *rho* correlation coefficient.

General Linear Model Univariate (GLM Univariate) was performed for each OS marker to evaluate whether muscle injury, anthropometric and metabolic parameters, physical activity, and AFC are predictors of circulating markers of OS. *p* < 0.05, 2-sided was considered for statistical significance.

## 3. Results

### 3.1. Characteristics of Participants

Clinical information, analysis of the *DMD* gene or dystrophin expression, and reading frame prediction of the *DMD* gene [28] were used to confirm the diagnosis of DMD in the twenty-eight patients (Table S1, Figure S1).

Ambulatory and non-ambulatory DMD patients were compared to demonstrate the difference in clinical characteristics between both groups. Age, Vignos scale score, GMFCS scale score, Brooke scale score, height, and body weight were significantly higher (*p* ≤ 0.004) in the non-ambulatory group than in the ambulatory group. Accordingly, the MRC scale score, levels of circulating muscle injury markers (CPK, ALT, and ALT), total cholesterol concentration, and physical activity score were significantly lower (*p* ≤ 0.008) in the non-ambulatory patients than in the ambulatory patients. The BMI percentile, glucose, triglycerides, VLDL-C, physical therapy, and frequency of AFC were similar between groups (*p* > 0.05) (Table 1).

### 3.2. Oxidative Stress in Ambulatory and Non-Ambulatory Patients

**Figure 1:**
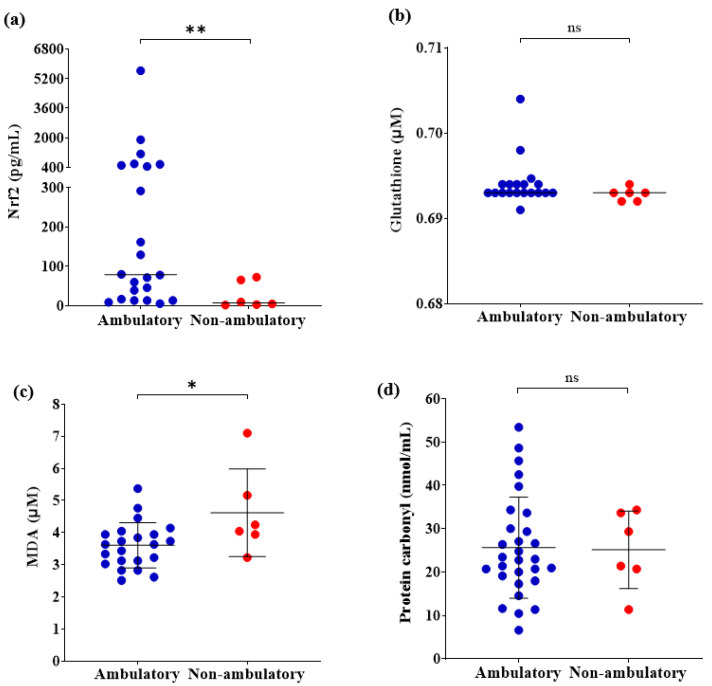
Comparison of circulating OS markers between ambulatory (*n* = 22) and non-ambulatory (*n* = 6) DMD patients. (**a**) Nrf2; (**b**) Glutathione (*n* = 19, *n* = 6); (**c**) MDA; (**d**) Protein carbonyl. Student *t*-test or Mann-Whitney *U*-test according to data distribution. * *p* ≤ 0.05, ** *p* ≤ 0.01. OS, Oxidative stress; Nrf2, NFE2 like bZIP transcription factor 2; MDA, Malondialdehyde; ns, not significative.

Regarding the circulating antioxidant markers, levels of Nrf2 were significantly lower in the non-ambulatory patients than in the ambulatory patients (*p ≤* 0.01) (Figure 1a). However, no differences were observed in levels of glutathione between both groups (*p* > 0.05) (Figure 1b). Concerning the oxidative damage markers, levels of MDA were higher in the non-ambulatory patients than in the ambulatory patients (*p ≤* 0.05) (Figure 1c). Nevertheless, there were no differences in protein carbonyl (*p* > 0.05) (Figure 1d).

### 3.3. Correlation of Circulating OS with Age and Muscle Injury Markers

**Figure 2:**
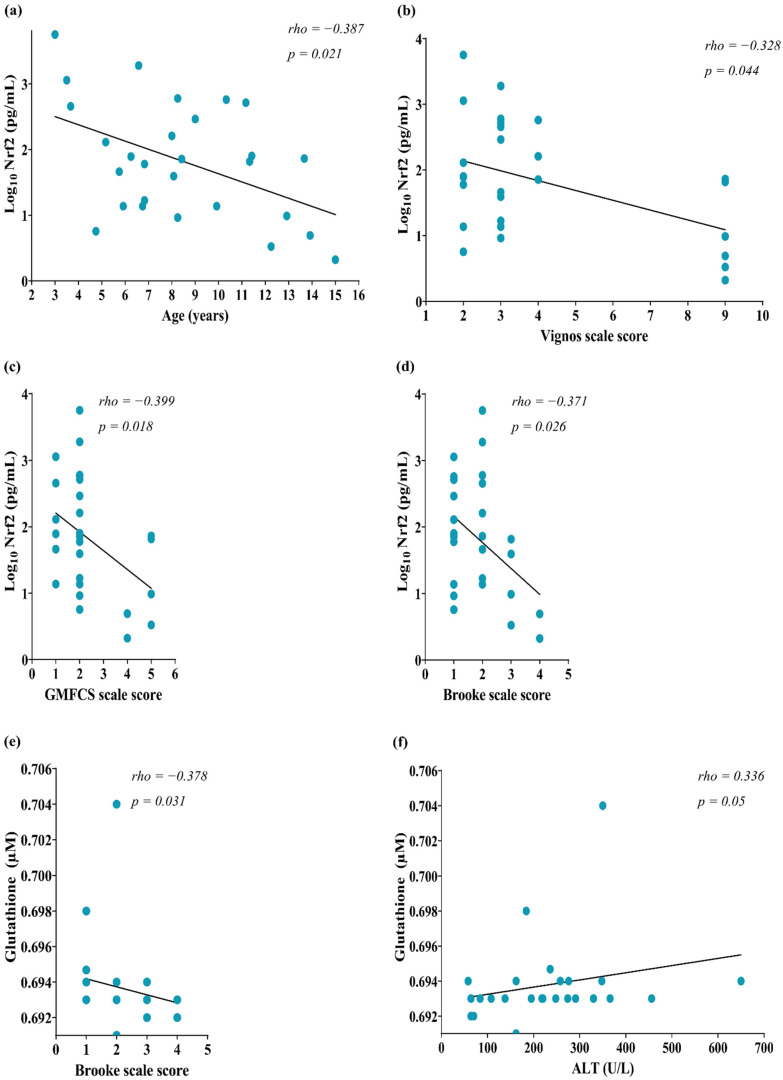

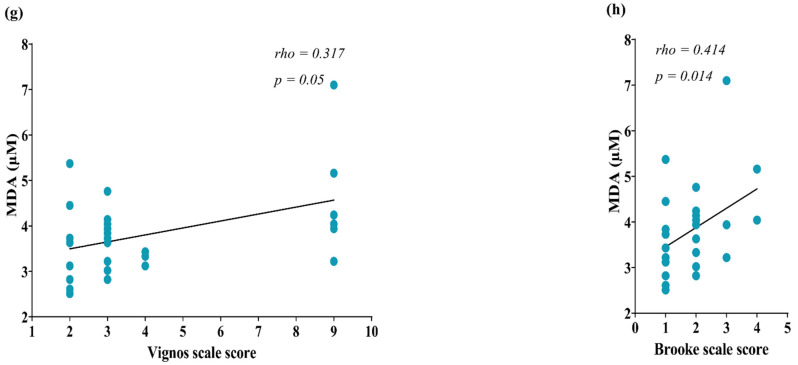
Correlation of circulating markers of OS with muscle injury parameters of DMD patients. (**a**) Nrf2 & GMFCS scale score; (**b**) Nrf2 & Vignos scale score; (**c**) NRF2 & GMFCS scale score; (**d**) Nrf2 & Brooke scale score; (**e**) Glutathione & Brooke scale score; (**f**) Glutathione & ALT; (**g**) MDA & Vignos scale score; (**h**) MDA & Brooke scale score. The correlation was computed by Spearman’s correlations (N = 28) for glutathione, N = 25. Data of Nrf2 concentration were transformed to Log_10_ for a better data representation in the figures. OS, Oxidative stress; Nrf2, NFE2 like bZIP transcription factor 2; GMFCS, Gross Motor Function Classification System; MDA, Malondialdehyde.

Correlation analyses were performed to know whether circulating OS was associated with a muscle injury. We identified a negative correlation between Nrf2 concentration and the age of patients (*rho* = −0.387, *p* = 0.021, Figure 2a). Also, Nrf2 negatively correlated with the Vignos scale score (*rho* = −0.328, *p* = 0.044, Figure 2b), GMFCS scale (*rho* = −0.399, *p* = 0.018, Figure 2c), and Brooke scale scores (*rho* = −0.371, *p* = 0.026, Figure 2d). Regarding glutathione, this marker correlated negatively with the Brooke scale score (*rho* = −0.378, *p* = 0.031, Figure 2e) and positively with ALT (*rho* = 0.336, *p* = 0.05, Figure 2f). However, we did not identify a correlation of AST with OS markers (*p* > 0.05, data not shown).

Concerning the markers of oxidative damage, MDA showed a correlation with Vignos (*rho* = 0.317, *p* = 0.05, Figure 2g) and Brooke scale scores (*rho* = 0.414, *p* = 0.014, Figure 2h). No correlations were found between the circulating markers of OS and MRC muscle strength, nor with the enzymatic markers of muscle injury CPK and AST (Table S2).

### 3.4. Correlation of Circulating OS with Anthropometric Parameters, Metabolic Indicators, Physical Activity Parameters, and Frequency of AFC

From all these clinical and dietary variables, we found a pair of correlations: circulating Nrf2 concentration correlated with height (*rho* = −0.396, *p* < 0.05), and MDA concentration correlated with cereals and tubers consumption (*rho* = 0.465, *p* < 0.05) as is showed in Table 2.

Additionally, we computed correlations among antioxidants and oxidative damage markers concentration. The results evidenced a significant correlation between glutathione and MDA concentration (*rho* = −0.399, *p* = 0.024). However, no correlation was found among the other OS markers (Table 3).

### 3.5. Predictors of Circulating Levels of OS Markers in DMD Patients

We generated three models with the GLM Univariate analyses (adjusted by covariables). Model 1 (*R^2^* = 0.248, *p* = 0.011) included age, GMFCS scale, and Brooke scale, which showed that age predicts the levels of Nrf2 concentrations. Model 2 (*R^2^* = 0.094, *p* < 0.001) included the Brooke scale, ALT, and MDA; and Model 3 (*R^2^* = 0.094, *p* = 0.334) included Vignos scale, Brooke scale, glutathione, and cereals and tubers; however, models 2 and 3 did not evidence predictors (Table 4).

## 4. Discussion

It is well known that OS plays a crucial role in DMD pathophysiology, as it has been reported in muscle tissue from mdx mice and DMD patients [10,12,14,15]; however, OS has been scarcely studied in circulation [11,13,16]. Thus, studying circulating markers of OS is crucial to know changes in DMD and understanding whether or not those circulating markers reflect OS in muscle because access to muscle tissue is limited and invasive, mainly in pediatric patients.

In this study, we evaluated Nrf2, glutathione, MDA, and protein carbonyl concentration in the circulation of DMD patients of a wide range of ages (3–15 years), including ambulatory and non-ambulatory subjects.

We are reporting for the first time the levels of Nrf2 in the plasma of DMD patients. Nrf2 in plasma has been reported in other diseases; for instance, patients with recent-onset type 2 diabetes [29]. We observed a lower level of this transcription factor in non-ambulatory DMD subjects. Nrf2, in response to OS, regulates the expression of antioxidant enzyme genes by interacting with the antioxidant-responsive element [30]. Our results agree with a previous report of our research group in a different cohort with fewer DMD patients, where the mRNA of NRF2 tended to be lower in non-ambulatory patients [11]. Those findings agree with the results reported by Petrillo et al. [10] in muscle biopsies. The authors observed a reduced expression of Nrf2 in muscle biopsies of older patients (2–9 years of age) compared with younger patients (0–2 years of age). Therefore, our results and the previous evidence suggest that the concentration of Nrf2 in circulation and muscle seems to have a similar pattern of change according to the disease severity. Thus, circulating Nrf2 concentration could reflect the levels of this transcription factor in the muscle of DMD patients. Our results suggest that circulating Nrf2 concentration decreases with age and muscle damage, which could indicate that antioxidant protection mediated by Nrf2 diminishes with disease severity.

Nrf2 correlated with height; however, it could have an indirect relation with age. The findings of this study showed that antioxidant markers did not correlate with anthropometric parameters, metabolic indicators, physical activity, and frequency of AFC. Those observations support that antioxidant markers are mainly related to muscle damage severity.

Our results about the levels of circulating glutathione showed a difference between ambulatory and non-ambulatory patients. Published information about glutathione in DMD is inconsistent; for instance, it was reported that glutathione concentration in muscle biopsies and whole blood in DMD patients (0–9 years of age) was lower than in healthy controls [10]. However, Burns and collaborators reported that glutathione concentration in the diaphragm of mdx mice (8-week-old) was similar to that of healthy mice [12]. Additionally, Petrillo et al. [10] observed that glutathione concentration was higher in older (0–2 vs. 2–9 years of age) DMD patients. However, in whole blood, there were no detected changes in concentrations [10].

Whether circulating glutathione reflects or not changes in this antioxidant molecule in muscle is still controversial. This affirmation is because we identified a correlation of glutathione concentration with the Brooke scale score but not with another scale of muscle function. The correlation of glutathione with ALT and Brooke scale score could be clues that glutathione relates to disease severity. However, more studies are needed because, as mentioned, the results are inconsistent with the other muscle injury indicators. We can not exclude that the low concentration observed in our outcomes could even result from glutathione depletion in muscle from DMD patients. However, this hypothesis must be tested.

Regarding MDA, we found that the concentration of this marker is higher in DMD patients with worst muscle function compared with those with better muscle function. This result agrees with a previous observation of our group research, where we reported that circulating concentrations of MDA and 8-isoprostane were higher in non-ambulatory DMD patients [11]. Also, in the present study, we found that Vignos and Brooke scale scores correlated with MDA concentration. Thus, our findings support the previously reported data and the hypothesis that circulating MDA concentration is associated with DMD progression. As we mentioned above for antioxidant markers, the absence of correlations between MDA and anthropometric parameters, metabolic indicators, physical activity, and frequency of AFC suggests that MDA is related to muscle damage severity.

Finally, protein carbonyl concentration was not different between ambulatory and non-ambulatory DMD patients, and any correlation with muscle function was evidenced. Ground et al. [16] reported that protein carbonyl concentrations were higher in the muscles of older mdx mice than in younger mdx mice. However, when they measured the circulating concentration of protein carbonyl, they did not find differences between young and old mice concluding that protein carbonyl is not a good circulating marker of protein oxidation in muscle. Thus, our results agree with Ground et al. [16], supporting the proposal that protein carbonyl is not a good marker in muscle or circulation of DMD patients.

When we analyzed the correlation between circulating antioxidants markers and circulating oxidative damage markers, we found a negative correlation between glutathione and MDA. The design of the study does not lead us to propose a mechanistic explanation for these results. However, several authors have reported the same antagonistic association between both markers [31,32,33,34].

The GLM Univariate analysis predicted that age is an important variable related to changes in circulating Nrf2 (β coefficient = −258.589), which interestingly support by the first time that changes in Nrf2 are reflected in circulation (*R*^2^ = 0.248, *p* = 0.011). Age is an indicator of disease severity because DMD is a degenerative disease. Thus, the GLM Univariate analysis evidenced that circulating Nrf2 changes the function of the disease severity.

We identified the following limitations of the study; small sample, lack of a healthy group to know control values, and cross-sectional design, which makes it difficult to determine a cause-effect relationship. Additionally, with the design of this study is not possible to know the exact origin of circulating markers. They could come from adipose tissue and thus account for the higher levels of oxidative damage in the non-ambulatory patients. Finally, our data do not entirely reflect oxidative damage in muscle tissue. Despite these limitations, this study has important strengths. Firstly, in this study, we included patients with a gradient of muscle damage. Therefore, patients with contrasting muscle functions were studied. Our outcomes evidence the importance of antioxidant response and oxidative damage in DMD pathophysiology. The findings in this work, together with previous information, highlight the importance of antioxidant-rich foods or nutritional antioxidant therapies in DMD patients from the early stages (ambulatory patients) of the disease to help to prevent the oxidative status as much as possible, improving muscle preservation and positively impacting the quality of life. Finally, this research opens a field of study to elucidate the mechanisms involved in OS by considering a panel of markers in dystrophic muscle for a better understanding of the physiopathology of this disease.

## 5. Conclusions

Our findings suggest that DMD patients with the worst muscle function have more significant oxidative damage and a lower antioxidant function than DMD patients with better muscle function. We also showed evidence that circulating markers such as Nrf2, glutathione, and MDA are modified during the severity of Duchenne disease. The outcomes also add new knowledge that circulating Nrf2 could be considered a good indicator of oxidative damage in muscle. Finally, we give evidence that protein carbonyl is not an adequate circulating marker of muscle damage in DMD.

## Figures and Tables

**Table 1:** Characteristics of ambulatory and non-ambulatory DMD patients.

	Ambulatory *n* = 22	Non-Ambulatory *n* = 6	*p*-Value
Age (year)	7.2 ± 2.4	13.2 ± 1.3	<0.001
Muscle injury markers			
Vignos scale (score)	3 (2, 4)	9 (9, 9)	<0.001
GMFCS scale (score)	2 (1, 2)	5 (4, 5)	<0.001
Brooke scale (score)	1 (1, 3)	3 (2, 4)	<0.001
MRC muscle strength (%)	91 (75, 100) ^†^	71 (43, 77) ^¶^	<0.001
CPK (U/L)	14,148 ± 6537	5842 ± 1601	<0.001
AST (U/L)	203 ± 67	98 ± 19	<0.001
ALT (U/L)	253 (84, 650)	67 (58, 138)	<0.001
Anthropometric parameters			
Height (cm)	113.92 ± 14.49	149.48 ± 14.09	<0.001
Body weight (kg)	19.13 (11.70, 48.80)	39.20 (22.20, 53.40)	0.004
BMI (percentile)	24 (1, 97)	6 (1, 77)	0.157
Metabolic indicators			
Glucose (mg/dL)	85 ± 7	87 ± 9	0.573
Triglycerides (mg/dL)	134 (56, 326)	120 (80, 290)	0.758
Total cholesterol (mg/dL)	155 (124, 264)	116 (108, 155)	0.002
VLDL-C (mg/dL)	27 (11, 65)	24 (16, 58)	0.758
Physical activity parameters			
Paq-C (score)	2.44 ± 0.82 ^‡^	1.46 ± 0.31	0.008
Physical therapy (s/w)	7 (0, 7)	1 (0, 2)	0.112
Frequency of AFC			
Vegetables	0.11 ± 0.08 ^‡^	0.12 ± 0.09	0.901
Fruits	0.07 (0, 0.28) ^‡^	0.12 (0.07, 0.58)	0.201
Legumes	0.05 (0, 0.68) ^‡^	0.03 (0.01, 0.05)	0.297
Cereals and tubers	0.23 ± 0.20 ^‡^	0.38 ± 0.18	0.102
Animal and vegetable fats	0.05 (0, 0.48) ^‡^	0.06 (0.05, 0.33)	0.175
Sugars and confectionery	0.07 (0, 0.89) ^‡^	0.07 (0, 0.22)	0.554
Beverages	0.24 (0, 0.72) ^‡^	0.43 (0.02, 1)	0.342

DMD, Duchenne muscular dystrophy; GMFCS, Gross Motor Function Classification System; MRC, Medical Research Council; CPK, Creatine phosphokinase; AST, Aspartate aminotransferase; ALT, Alanine aminotransferase; BMI, Body mass index; VLDL-C, Very low-density lipoprotein-cholesterol; s/w, Sessions/week; AFC, Antioxidant food consumption. Data are shown as mean ± standard deviation (SD) or median (minimum, maximum). Student *t*-test or Mann-Whitney *U*-test according to data distribution. ^†^ *n* = 21, ^‡^ *n* = 20, ^¶^ *n* = 5.

**Table 2:** Correlation of circulating markers of OS with anthropometric parameters, metabolic indicators, physical activity parameters, and frequency of AFC of DMD patients.

	Antioxidants Markers		Oxidative Damage Markers	
	Nrf2 (pg/mL) (N = 28)	Glutathione (µM) (N = 25)	MDA (µM) (N = 28)	P. Carb. (nmol/mL) (N = 28)
Anthropometric parameters				
Height (cm)	−0.396 *	−0.156	0.324	0.011
Body weight (kg)	−0.355	−0.216	0.328	−0.028
BMI (percentile)	−0.014	−0.242	0.182	−0.286
Metabolic indicators				
Glucose (mg/dL)	0.136	−0.051	0.258	0.038
Triglycerides (mg/dL)	−0.026	−0.020	−0.060	0.168
Total cholesterol (mg/dL)	0.183	0.136	−0.269	−0.078
VLDL-C (mg/dL)	−0.026	−0.020	−0.060	0.168
Physical activity parameters				
Paq-C (score) ^†^	0.154	0.112	−0.283	0.041
Physical therapy (s/w)	−0.017	0.087	−0.070	−0.264
Frequency of AFC ^†^				
Vegetables	−0.002	0.068	0.033	0.119
Fruits	0.128	−0.157	0.292	−0.095
Legumes	−0.071	0.136	0.092	−0.240
Cereals and tubers	−0.215	−0.134	0.465 *	−0.065
Animal and vegetable fats	−0.324	−0.348	0.165	−0.083
Sugars and confectionery	−0.171	−0.045	−0.055	0.071
Beverages	−0.043	−0.080	−0.142	0.137

Correlation coefficients were calculated by Spearman correlation (N = 28). OS, Oxidative stress; AFC, Antioxidant food consumption; BMI, Body mass index; HOMA-IR, Homeostasis Model Assessment-Insulin Resistance; VLDL-C, Very low-density lipoprotein-cholesterol; Paq-C, Physical activity questionnaire-C; s/w, Sessions/week; Nrf2, NFE2 like bZIP transcription factor 2; MDA, Malondialdehyde; P. Carb, Protein carbonyl. * *p* ≤ 0.05. ^†^ N = 26. The value of N changes in the frequency of AFC because data were unavailable.

**Table 3:** Correlation matrix between circulating antioxidants markers and circulating oxidative damage markers in DMD patients.

Markers	Nrf2	Glutathione ^†^	MDA	P. carb.
Nrf2	/	/	/	/
Glutathione ^†^	*rho* = 0.121 *p* = 0.282	/	/	/
MDA	*rho* = −0.242 *p* = 0.108	*rho* = −0.399 *p* = 0.024	/	/
P. carb.	*rho* = −0.057 *p* = 0.356	*rho* = −0.109 *p* = 0.302	*rho* = −0.241 *p* = 0.109	/

Correlation coefficients were calculated by Spearman correlation (N = 28). Nrf2, NFE2 like bZIP transcription factor 2; MDA, Malondialdehyde; P. carb., Protein carbonyl. ^†^ N = 25. The value of N changes in glutathione because plasma volume was not enough to measure the molecule.

**Table 4:** GLM Univariate analyses to evaluate predictors of circulating markers of OS in DMD patients.

Predictors	β Coefficient	Standard Error	T	*p*-Value
Model 1: Nrf2 *R*^2^ = 0.248, *p* = 0.011				
Age (years)	−258.589	97.201	−2.660	0.014
GMFCS scale (score)	363.948	281.133	1.295	0.208
Brooke scale (score)	74.802	275.685	0.271	0.788
Model 2: Glutathione *R*^2^ = 0.094, *p <* 0.001				
Brooke scale (score)	−7.927 × 10^−5^	0.001	−0.126	0.901
ALT (U/L)	2.986 × 10^−6^	4.042 × 10^−6^	0.739	0.468
MDA (µM)	−0.001	0.001	−0.881	0.388
Model 3: MDA *R*^2^ = 0.235, *p* = 0.334				
Vignos scale (score)	0.089	0.123	0.724	0.478
Brooke scale (score)	0.084	0.372	0.224	0.825
Glutathione (µM)	−73.826	78.881	−0.936	0.362
Cereals and tubers	0.652	1.122	0.581	0.569

GLM Univariate, General Linear Model Univariate; Nrf2, NFE2 like bZIP transcription factor 2; GMFCS, Gross Motor Function Classification System; ALT, Alanine aminotransferase; MDA, Malondialdehyde.

## Data Availability

The data are contained within the article and supplementary materials.

## Supplementary Materials

The following supporting information can be downloaded at: https://www.mdpi.com/article/10.3390/antiox12040871/s1, Table S1: Diagnostic and clinical characteristics of DMD patients; Figure S1: Pathogenic variants in the *DMD* gene of the study population; Table S2. Correlation of circulating markers of OS with age and muscle injury of DMD patients.

## References

[1] Rodríguez-Cruz M., Almeida-Becerril T., Atilano-Miguel S., Cárdenas-Conejo A., Bernabe-García M. (2020). Natural History of Serum Enzyme Levels in Duchenne Muscular Dystrophy and Implications for Clinical Practice. Am. J. Phys. Med. Rehabil..

[2] Mah J.K., Korngut L., Dykeman J., Day L., Pringsheim T., Jette N. (2014). A Systematic Review and Meta-Analysis on the Epidemiology of Duchenne and Becker Muscular Dystrophy. Neuromuscul. Disord..

[3] Guiraud S., Aartsma-Rus A., Vieira N.M., Davies K.E., van Ommen G.J.B., Kunkel L.M. (2015). The Pathogenesis and Therapy of Muscular Dystrophies. Annu. Rev. Genom. Hum. Genet..

[4] Allen D.G., Whitehead N.P., Froehner S.C. (2016). Absence of Dystrophin Disrupts Skeletal Muscle Signaling: Roles of Ca^2+^, Reactive Oxygen Species, and Nitric Oxide in the Development of Muscular Dystrophy. Physiol. Rev..

[5] Cruz-Guzmán O.D.R., Rodríguez-Cruz M., Escobar Cedillo R.E. (2015). Systemic Inflammation in Duchenne Muscular Dystrophy: Association with Muscle Function and Nutritional Status. Biomed. Res. Int..

[6] Kourakis S., Timpani C.A., de Haan J.B., Gueven N., Fischer D., Rybalka E. (2021). Targeting Nrf2 for the Treatment of Duchenne Muscular Dystrophy. Redox Biol..

[7] Betteridge D.J. (2000). What is oxidative stress?. Metabolism.

[8] Rando T.A., Disatnik M.H., Yu Y., Franco A. (1998). Muscle Cells from Mdx Mice Have an Increased Susceptibility to Oxidative Stress. Neuromuscul. Disord..

[9] Tebay L.E., Robertson H., Durant S.T., Vitale S.R., Penning T.M., Dinkova-Kostova A.T., Hayes J.D. (2015). Mechanisms of Activation of the Transcription Factor Nrf2 by Redox Stressors, Nutrient Cues, and Energy Status and the Pathways through Which It Attenuates Degenerative Disease. Free Radic. Biol. Med..

[10] Petrillo S., Pelosi L., Piemonte F., Travaglini L., Forcina L., Catteruccia M., Petrini S., Verardo M., D’Amico A., Musarò A. (2017). Oxidative Stress in Duchenne Muscular Dystrophy: Focus on the NRF2 Redox Pathway. Hum. Mol. Genet..

[11] Almeida-Becerril T., Rodríguez-Cruz M., Sánchez-González J.R., Villaldama-Soriano M.A., Atilano-Miguel S., Villa-Morales J., Cárdenas-Conejo A., Cárdenas-Vázquez R. (2021). Circulating markers of oxidative stress are associated with a muscle injury in patients with muscular dystrophy Duchenne. Brain Dev..

[12] Burns D.P., Drummond S.E., Bolger D., Coiscaud A., Murphy K.H., Edge D., O’Halloran K.D. (2019). N-Acetylcysteine Decreases Fibrosis and Increases Force-Generating Capacity of Mdx Diaphragm. Antioxidants.

[13] Grosso S., Perrone S., Longini M., Bruno C., Minetti C., Gazzolo D., Balestri P., Buonocore G. (2008). Isoprostanes in Dystrophinopathy: Evidence of Increased Oxidative Stress. Brain Dev..

[14] Hunter M.I., Mohamed J.B. (1986). Plasma Antioxidants and Lipid Peroxidation Products in Duchenne Muscular Dystrophy. Clin. Chim. Acta.

[15] Mechler F., Imre S., Dioszeghy P. (1984). Lipid Peroxidation and Superoxide Dismutase Activity in Muscle and Erythrocytes in Duchenne Muscular Dystrophy. J. Neurol. Sci..

[16] Al-Mshhdani B.A., Grounds M.D., Arthur P.G., Terrill J.R. (2021). A Blood Biomarker for Duchenne Muscular Dystrophy Shows That Oxidation State of Albumin Correlates with Protein Oxidation and Damage in Mdx Muscle. Antioxidants.

[17] Carraro E., Schilirò T., Biorci F., Romanazzi V., Degan R., Buonocore D., Verri M., Dossena M., Bonetta S., Gilli G. (2018). Physical Activity, Lifestyle Factors and Oxidative Stress in Middle Age Healthy Subjects. Int. J. Environ. Res. Public Health.

[18] Almeida-Becerril T., Rodríguez-Cruz M., Hernández-Cruz S.Y., Ruiz-Cruz E.D., Mendoza C., Cárdenas-Conejo A., Escobar-Cedillo R.E., Ávila-Moreno F., Aquino-Jarquin G. (2022). Natural history of circulating miRNAs in Duchenne disease: Association with muscle injury and metabolic parameters. Acta Neurol. Scand..

[19] Vignos P.J., Archibald K.C. (1960). Maintenance of Ambulation in Childhood Muscular Dystrophy. J. Chronic Dis..

[20] Palisano R., Rosenbaum P., Walter S., Russell D., Wood E., Galuppi B. (1997). Development and Reliability of a System to Classify Gross Motor Function in Children with Cerebral Palsy. Dev. Med. Child Neurol..

[21] Brooke M.H., Griggs R.C., Mendell J.R., Fenichel G.M., Shumate J.B., Pellegrino R.J. (1981). Clinical Trial in Duchenne Dystrophy. I. The Design of the Protocol. Muscle Nerve.

[22] Compston A. (2010). Aids to the investigation of peripheral nerve injuries. Medical Research Council: Nerve Injuries Research Committee. His Majesty’s Stationery Office: 1942; pp. 48 (iii) and 74 figures and 7 diagrams; with aids to the examination of the peripheral nervous system. By Michael O’Brien for the Guarantors of Brain. Saunders Elsevier: 2010; pp. [8] 64 and 94 Figures. Brain.

[23] Hata Y., Nakajima K. (1986). Application of Friedewald’s LDL-Cholesterol Estimation Formula to Serum Lipids in the Japanese Population. Jpn. Circ. J..

[24] Kowalski K.C., Crocker P.R., Donen R.M. (2004). The Physical Activity Questionnaire for Older Children (PAQ-C) and Adolescents (PAQ-A) Manual.

[25] ENSANUT. https://ensanut.insp.mx/.

[26] Zotor F., Sheehy T., Lupu M., Kolahdooz F., Corriveau A., Sharma S. (2012). Frequency of Consumption of Foods and Beverages by Inuvialuit Adults in Northwest Territories, Arctic Canada. Int. J. Food Sci. Nutr..

[27] Carlsen M.H., Halvorsen B.L., Holte K., Bøhn S.K., Dragland S., Sampson L., Willey C., Senoo H., Umezono Y., Sanada C. (2010). The Total Antioxidant Content of More than 3100 Foods, Beverages, Spices, Herbs and Supplements Used Worldwide. Nutr. J..

[28] Leiden Muscular Dystrophy Pages. https://www.dmd.nl/.

[29] Sireesh D., Dhamodharan U., Ezhilarasi K., Vijay V., Ramkumar K.M. (2018). Association of NF-E2 Related Factor 2 (Nrf2) and inflammatory cytokines in recent onset Type 2 Diabetes Mellitus. Sci. Rep..

[30] Itoh K., Chiba T., Takahashi S., Ishii T., Igarashi K., Katoh Y., Oyake T., Hayashi N., Satoh K., Hatayama I. (1997). An Nrf2/Small Maf Heterodimer Mediates the Induction of Phase II Detoxifying Enzyme Genes through Antioxidant Response Elements. Biochem. Biophys. Res. Commun..

[31] Orman A., Kahraman A., Çakar H., Ellidokuz H., Serteser M. (2005). Plasma Malondialdehyde and Erythrocyte Glutathione Levels in Workers with Cement Dust-Exposure Silicosis. Toxicology.

[32] Kasperczyk A., Słowińska-Łożyńska L., Dobrakowski M., Zalejska-Fiolka J., Kasperczyk S. (2014). The Effect of Lead-Induced Oxidative Stress on Blood Viscosity and Rheological Properties of Erythrocytes in Lead Exposed Humans. Clin. Hemorheol. Microcirc..

[33] Shraideh Z., Badran D., Hunaiti A., Battah A. (2018). Association between Occupational Lead Exposure and Plasma Levels of Selected Oxidative Stress Related Parameters in Jordanian Automobile Workers. Int. J. Occup. Med. Environ. Health.

[34] Tualeka A.R., Martiana T., Ahsan A., Russeng S.S., Meidikayanti W. (2019). Association between Malondialdehyde and Glutathione (L-Gamma-Glutamyl-Cysteinyl-Glycine/GSH) Levels on Workers Exposed to Benzene in Indonesia. Open Access Maced. J. Med. Sci..

