# Supplementary material for: Circulating Nrf2, Glutathione, and Malondialdehyde Correlate with Disease Severity in Duchenne Muscular Dystrophy
Source: Antioxidants (Basel). 2023 Apr 3;12(4):871. doi: 10.3390/antiox12040871 (PMC10135301; doi:10.3390/antiox12040871)
Supplement: Supplementary file 1 [file antioxidants-12-00871-s001.zip › antioxidants-2237741-supplementary.pdf]

Circulating Nrf2, glutathione, and malondialdehyde correlate with disease severity in Duchenne muscular dystrophy

Tomas Almeida-Becerril, Maricela Rodríguez-Cruz, Judith Villa-Morales, Christian Ricardo Sánchez Mendoza, Jose Emilio Galeazzi-Aguilar

Supplementary Material

Table S1. Diagnostic and clinical characteristics of DMD patients.

Patient	Age (years) at			Calf pseudo-hypertrophy	Gowers' sign	CPK (U/L)
	First symptoms	Loss of ambulation	Study day			
1	1	Ambulant	3.7	Yes	Yes	14300
2	2	Ambulant	3.0	NR	Yes	20752
3	4	Ambulant	4.8	Yes	Yes	14522
4	2	Ambulant	6.3	NR	Yes	23352
5	<1	Ambulant	5.9	Yes	Yes	15531
6	3	Ambulant	5.2	Yes	Yes	7784
7	2	Ambulant	3.5	Yes	Yes	12213
8	3	Ambulant	6.8	Yes	NR	20100
9	3	Ambulant	5.8	NR	NR	10736
10	<1	Ambulant	6.6	NR	Yes	15670
11	4	11	15.0	Yes	Yes	14265
12	4	11	13.9	Yes	Yes	6650
13	4	Ambulant	8.1	NR	Yes	8668
14	3	Ambulant	6.8	Yes	Yes	18827
15	<1	10	11.3	NR	Yes	8951
16	4	Ambulant	9.9	Yes	Yes	9028
17	<1	Ambulant	8.3	Yes	Yes	11741
18	2	13	13.7	NR	NR	18601
19	7	Ambulant	11.2	Yes	Yes	17022
20	3	Ambulant	8.4	Yes	Yes	14334
21	4	11	12.3	Yes	NR	5380
22	4	11	12.9	Yes	NR	32346
23	2	Ambulant	11.4	Yes	Yes	9140
24	5	Ambulant	8.3	Yes	Yes	13850
25	3	Ambulant	10.3	Yes	Yes	14567
26	3	Ambulant	8.0	Yes	Yes	14625
27	1	Ambulant	6.8	Yes	Yes	20550
28	3	Ambulant	9.0	Yes	Yes	7500

DMD, Duchenne muscular dystrophy; CPK, creatine phosphokinase; NR, not registered.

Patients		Exonic sequence of *DMD* gene (1–8, 9–63, 64–69, 70–79)	Reading frame
P1	Amb.	Del_48,50 (mPCR)	Frameshift
P2	Amb.	Del_52 (mPCR)	Frameshift
P3	Amb.	Del_45-47,50-52 (MLPA)	Frameshift
P4	Amb.		*
P5	Amb.	Del 1-67 (Sec.)	Frameshift
P6	Amb.	Del_50 (mPCR)	Frameshift
P7	Amb.	Del_48,50 (mPCR)	Frameshift
P8	Amb.		*
P9	Amb.	Dup_46-61 (MLPA)	Frameshift
P10	Amb.		*
P11	No amb.	Del_51-52 (mPCR)	In-frame
P12	No amb.	Del_51-52 (mPCR)	In-frame
P13	Amb.	Del_45-52 (MLPA)	Frameshift
P14	Amb.	Del_48,50-52 (mPCR)	Frameshift
P15	No amb.		*
P16	Amb.	Del_51 (MLPA)	Frameshift
P17	Amb.	Del_53-55 (MLPA)	Frameshift
P18	No amb.	Del_44 (mPCR)	Frameshift
P19	Amb.	Del_48,50 (mPCR)	Frameshift
P20	Amb.	Del_45-52 (MLPA)	Frameshift
P21	No amb.	Del_51-53 (MLPA)	Frameshift
P22	No amb.	Del_45, 48, 50-52 (mPCR)	Frameshift
P23	Amb.	Del_45 (mPCR)	Frameshift
P24	Amb.	Del_46-51 (MLPA)	Frameshift
P25	Amb.	Del_10,11 (MLPA)	Frameshift
P26	Amb.	Del_48-52 (MLPA)	Frameshift
P27	Amb.	Del_50-54 (MLPA)	Frameshift
P28	Amb.	Del_50-52 (MLPA)	Frameshift

Figure S1. Pathogenic variants in the *DMD* gene of the study population. Amb, ambulatory; mPCR, PCR multiplex; MLPA, Multiplex Ligation-dependent Probe Amplification; Seq, Sequenciation; Del, Deletion; Dup, Duplication.

—— Deletion —— Duplication * Absence of dystrophin (muscle biopsy)

Table S2. Correlation of circulating markers of OS with age and muscle injury of DMD patients.

	**Antioxidants markers**		**Oxidative damage markers**	
	Nrf2 (pg/mL) (N = 28)	Glutathione (μM) (N = 25)	MDA (μM) (N = 28)	P. Carb. (nmol/mL) (N = 28)
Age (year)	-0.387*	-0.138	0.244	0.049
Muscle injury parameters				
Vignos scale (score)	-0.328*	-0.216	0.317*	0.228
GMFCS scale (score)	-0.399*	-0.154	0.307	-0.119
Brooke scale (score)	-0.371*	-0.378*	0.414*	0.068
MRC muscle strength (%) †	0.261	0.009	-0.293	-0.197
CPK (U/L)	0.105	0.158	-0.137	-0.087
AST (U/L)	0.135	0.290	-0.266	0.053
ALT (U/L)	0.168	0.336*	-0.263	0.070

Correlation coefficients were calculated by Spearman correlation (N = 28).

OS; Oxidative stress; GMFCS, Gross Motor Function Classification System; MRC, Medical Research Council; CPK, Creatine phosphokinase; AST, Aspartate aminotransferase; ALT, Alanine aminotransferase; Nrf2, NFE2 like bZIP transcription factor 2; MDA, Malondialdehyde; P. Carb, Protein carbonyl.

* $p \leq 0.05$, ** $p \leq 0.01$.

† N = 26.

The value of N changes in some variables because some data were unavailable.
